# Three Arguments Against Institutional Conscientious Objection, and Why They Are (Metaphysically) Unconvincing

**DOI:** 10.1093/jmp/jhae012

**Published:** 2024-04-01

**Authors:** Xavier Symons, Reginald Mary Chua

**Affiliations:** Human Flourishing Program in the Institute for Quantitative Social Science, Harvard University, Cambridge, MA, USA; University of Notre Dame Australia, Fremantle, WA, Australia

**Keywords:** abortion, agency, conscience, conscientious objection, euthanasia, healthcare, institutions

## Abstract

The past decade has seen a burgeoning of scholarly interest in conscientious objection in healthcare. While the literature to date has focused primarily on individual healthcare practitioners who object to participation in morally controversial procedures, in this article we consider a different albeit related issue, namely, whether publicly funded healthcare institutions should be required to provide morally controversial services such as abortions, emergency contraception, voluntary sterilizations, and voluntary euthanasia. Substantive debates about institutional responsibility have remained largely at the level of first-order ethical debate over medical practices which institutions have refused to offer; in this article, we argue that more fundamental questions about the metaphysics of institutions provide a neglected avenue for understanding the basis of institutional conscientious objection. To do so, we articulate a metaphysical model of institutional conscience, and consider three well-known arguments for undermining institutional conscientious objection in light of this model. We show how our metaphysical analysis of institutions creates difficulties for justifying sanctions on institutions that conscientiously object. Thus, we argue, questions about the metaphysics of institutions are deserving of serious attention from both critics and defenders of institutional conscientious objection.

## I. INTRODUCTION

The past decade has seen a burgeoning of scholarly interest in conscientious objection in healthcare. Commentators have focused in particular on whether individual medical practitioners should have the right to refuse to participate in the provision of morally controversial medical procedures. Ethicists and policymakers have expressed concern that objecting doctors may impede patients’ access to services such as abortion, emergency contraception, or euthanasia (Savulescu, 2006, 297). Some ethicists argue that doctors have a professional duty to provide patients with services that are safe, legal, and professionally sanctioned. Proponents of conscience rights, in contrast, argue that broad conscience protections—both in private and public life—are an important part of a well-functioning liberal democracy. The doctors’ right to act on their conscience should not, on this view, be subordinated in favor of a dominant conception of morality or professional practice (Sulmasy, 2017).

While the literature to date has focused primarily on individual healthcare practitioners who object to participation in morally controversial procedures, in this article, we consider a different, albeit related issue, namely, whether publicly funded healthcare institutions should be required to provide morally controversial services such as abortions, emergency contraception, voluntary sterilizations, and voluntary euthanasia. The issue of institutional conscientious objection (ICO) is already a pressing question, because publicly funded religiously affiliated healthcare providers in countries such as Australia, the United States, and Canada refuse to provide many procedures that are legal and sanctioned by relevant professional associations. ICO will be of even greater importance in the future, if the current growth trends of religiously affiliated healthcare providers continue (Hafner, 2018).

For argument’s sake, we presume that services such as euthanasia and abortion are legal and are sanctioned by relevant professional associations.^1^ Our aim is to critique three common arguments that are leveled at institutions that object to the provision of morally controversial procedures. We do not do this by contributing to first-order debates over the ethics of practices such as abortion and euthanasia, despite the real impact these debates have on questions of conscientious objection. Rather, our procedure is to show that arguments against ICO are also impacted by assumptions about the metaphysics of institutions, and that questions about the metaphysics of institutional agency require further attention before ethical questions about ICO can be properly answered. To do so, we begin by proposing a model for an account of healthcare institutions as group agents and bearers of conscience. In light of this model, we critically evaluate three arguments against ICO. These arguments, analysis of which occupies the bulk of the paper, can be put briefly as follows. First, institutions do not have a conscience, and therefore need not be afforded conscience protections. Second, healthcare institutions that receive public funding should be obliged to provide comprehensive access to services that are safe and legal, and thus cannot be afforded conscience protections insofar as these protections conflict with such an obligation. Third, accommodating ICO can limit access to essential services when the institutions in question are sole healthcare providers in a given region; in light of this, institutions should not be afforded conscientious objection protections.

These arguments appear to offer independent, “publicly neutral” grounds for undermining ICO, which thus appear especially appropriate for consideration by liberal states in deliberations over healthcare funding and regulation. In this paper, we show that, despite their apparent promise, these arguments are unconvincing: they either neglect key features of the nature and practice of institutions that typically invoke ICO, or else they must tacitly invoke contentious assumptions about the nature of institutional conscience and institutional agency that conflict with the model of institutional agency presented in this paper. Thus, if the model put forward in this paper is metaphysically coherent, these arguments fail to demonstrate why institutions have an obligation to provide healthcare services that are in opposition to their mission and values.^2^

It is our hope that this article promotes further critical reflection on this topic among policymakers and the academic community. There has been widespread criticism of religious institutions that refuse to provide morally controversial procedures (Nelson, 2018). Yet, we believe that when the arguments at the center of these criticisms and their underlying assumptions about the nature of institutions are subject to scrutiny, it becomes more difficult to justify sanctions on institutions that conscientiously object.

## II. OVERVIEW

ICO refers to the refusal of healthcare institutions to provide medical services that violate the institution’s ethical standards or mission. The right of healthcare institutions to object conscientiously is protected in law in many jurisdictions, in addition to being sanctioned by medical associations. US law, for example, recognizes the right of healthcare institutions to refuse to participate in the provision of abortions and voluntary sterilizations. The “Church Amendments”—conscience-based statutes enacted federally in 1973—state that public funding for religious healthcare facilities should not be conditional on the provision of these services (Bertelsen, 2013). Australian abortion law, in contrast, is silent on the question of ICO, neither prohibiting or allowing ICO (Haining et al., 2022, 254–255). While specific law on conscientious objection differs in E.U. member states, the Council of Europe passed a resolution in 2010 stating that “no person, hospital or institution shall be coerced, held liable or discriminated against in any manner because of a refusal to perform, accommodate, assist, or submit to an abortion” (Council of Europe, 2010).

Some medical associations officially recognize the right of institutions to refuse to participate in medical procedures that are contrary to the ethos of the organization. For example, the Australian Medical Association recognizes ICO as a legitimate form of conscientious objection, though it notes that institutions should notify the public of the services that they do not provide (Australian Medical Association, 2019). Other medical associations, such as the American Medical Association, have no official position on the issue of ICO. To our knowledge, the American Medical Association has been completely silent about the nonparticipation of religiously affiliated healthcare providers in the provision of services such as abortion. The Association remains officially opposed to the practice of voluntary euthanasia, and in this respect is in agreement with the position of religiously affiliated healthcare institutions on the provision of euthanasia (American Medical Association, 2019).

Yet, ICO has been subject to extensive criticism both in academia as well as in the realm of public policy. Some of these criticisms stem from familiar concerns having to do with the morality of controversial practices such as abortion and euthanasia. For instance, Fiala and Arthur write,

[a fetus] has only the potential to become a person—it is still an inseparable and fully dependent part of a woman’s body and not an individual human being . . . Abortion and contraception preserve the health and lives of women, while those practicing [conscientious objection] put women’s lives at risk. (2014, 15)

Similarly, Savulescu and Schuklenk write:

most people who believe contraception, abortion and euthanasia are wrong don’t believe they are evil in the same way as, for example, torture or genocide are evil. If its rightness or wrongness is of a type or degree that it is a matter of personal preference (ethical relativism), it should not have an impact on patient care. (2017, 167)

It is worth noting that these arguments do not simply take a stand on the ethical permissibility of conscientious objection. They additionally involve implicit controversial “first-order” ethical commitments: for instance, the belief that destruction of a fetus is not morally wrong, or the belief that euthanasia is at best a matter of personal preference, and not comparable to matters of ethical obligation (such as the obligation not to murder or commit genocide).

Some have argued—rightly so, in our opinion—that arguments against conscientious objection premised controversial ethical commitments of this sort can involve problematic assumptions about the nature and scope of reasonable disagreement (Oderberg, 2018). While more could be said on this issue, we mention this feature of arguments against ICO simply to highlight the way in which debate surrounding ICO has tended to privilege first-order ethical concerns. As a result, questions of a more fundamental nature concerning the metaphysics of institutions have received relatively little attention.^3^ In this paper, we argue the contrary view: a closer consideration of the metaphysics of institutions leads to new avenues for reevaluating arguments surrounding ICO.

To make this case, we begin by briefly articulating a metaphysical model of institutional conscience based on Deborah Tollefsen’s account of group agency, before turning to consider three well-known arguments for undermining ICO in light of this model. First, some have argued that institutions do not have a conscience, and therefore need not be afforded conscience protections. Second, some have argued that healthcare institutions receiving public funding should be obliged to provide comprehensive access to services that are safe and legal, while third, yet others argue that ICO can give rise to morally problematic situations when the institutions in question are sole healthcare providers for a given region (especially in remote areas). We argue that, given the metaphysical analysis of institutions we offer, reasonable responses to these objections are available to the defender of ICO.

It is worth emphasizing at the outset that in this article we do not intend to argue for the truth of the metaphysical model we outline. Our primary goal is to illustrate how ongoing controversies in the metaphysics of institutions are deserving of serious attention from both critics and defenders of ICO. To achieve this goal, it is enough that the metaphysical model we articulate provides a coherent basis for the responses to the arguments against ICO we outline in this paper. Defenders of ICO who accept our arguments may draw the further conclusion that our model of institutions and institutional conscience is worth endorsing, precisely because of its ability to provide reasons for rejecting certain critiques of ICO. On the other hand, critics of ICO might draw the opposite conclusion, by seeing the links between our model and a robust defence of ICO as itself reason to look elsewhere for other models of institutional conscience. To draw either of these conclusions would be to bolster our argument that questions concerning the metaphysics of institutions are deserving of serious attention from participants in the debate over ICO.

Having clarified the aims of this article, we now turn to outlining a metaphysics of institutions and institutional conscience.

## III. A METAPHYSICS OF INSTITUTIONS AND INSTITUTIONAL CONSCIENCE

As mentioned above, our purpose in this section is not to endorse a particular metaphysics of institutions or group agency, in part because the issues in question are hotly contested, with ongoing debates between so-called methodological individualism and holism in the social sciences and debates about the nature of group agency (Tuomela, 2013; Zahle, 2016; Miller, 2022). However, in this section, we outline a realist metaphysics of institutions and institutional conscience, in part because criticisms of ICO often rest (explicitly or implicitly) on the assumption that institutions are not agents as such, and thus differ from individuals in failing to literally have a conscience. However, the question of whether institutions are agents is in fact a moot point, and some theorists regard institutional groups as possessing features that do in fact confer agency on them. To elaborate, it is instructive to consider Deborah Tollefsen’s interpretivist view of group agency.^4^ Tollefsen does not explicitly provide an account of institutional conscience; one task of this section is to extend Tollefsen’s account in that direction.

Tollefsen’s metaphysical account of groups is based on her interpretivist account of *agency* and of *intentional attitudes*. We briefly consider each issue in turn. According to Tollefsen, whether a given entity counts as an agent depends on the interpretive practices we would ideally employ in order to understand that entity. Following Daniel Dennett (1987), one key practice Tollefsen identifies is the *intentional stance*, that is, a mindset by which we seek to understand something “by treating it as if it were a rational agent whose actions are governed by its beliefs, intentions, and desires” (2015, 98). Along with other mindsets (e.g., the physical stance or the design stance), the intentional stance can in theory be applied to any entity, even an inanimate object like a flowerpot (Tollefsen, 2015).^5^ However, in practice, we regard the intentional stance as ideally restricted to contexts in which such a practice brings distinctive explanatory power. Tollefsen’s interpretivism takes as its premise the trustworthiness of our interpretive practices:

Interpretivism starts with our practice. It takes as its starting point the explanatory power of this practice. If we are able to understand and predict the behavior of a system using the intentional stance, then we have every reason to believe we are dealing with one. (2015, 103)

One consequence of Tollefsen’s interpretivism is that any entity to which we ideally apply the intentional stance counts as an agent: “if we can successfully make sense of another being—understand and interpret its behavior by using our folk psychology—it is an intentional agent” (2015, 97). Tollefsen’s account is noteworthy, in that it does not treat agency as based on an unobservable feature intrinsic to agents (e.g., an immaterial soul), but rather as based on how reasonable observers understand the way agents interact with their environment. Interpretivism, Tollefsen argues, yields the conclusion that agency is possessed not only by individual human beings but also by a wide variety of groups (e.g., the nation-state of Israel), since in each case we use the intentional stance to understand and predict their behavior.

Since the activity of agents is usually accompanied by intentional attitudes (e.g., beliefs, desires, etc.), Tollefsen also provides a closely related dispositional account of intentional attitudes:

According to interpretivism, mental states are not states of the head or brain but states of the whole agent or system. They are dispositional states in that they are defined in terms of what an agent will do, say, and think under certain circumstances. To believe that it is snowing is to be disposed to behave in certain ways under certain conditions and to form other intentional states related to those involving snow. (2015, 103)

What is important to note, for present purposes, is that this account parallels Tollefsen’s account of agency in being “observation-based”: whether an entity has mental states is something that be identified simply through observing its interactions with its environment. This allows for intentional attitudes to be ascribed not only to individuals (e.g., “John believes that it is snowing”) but also to groups in certain contexts (e.g., “Israel is convinced that Iran is secretly working towards developing a nuclear warhead”) (Gardner, 2021), that is, contexts where we engage in understanding them using the intentional stance and make inferences about how they are intentionally disposed to behave.

Needless to say, Tollefsen’s interpretivism has had its fair share of critics (cf. Strohmaier, 2020). However, as mentioned above, our purpose here is not to defend the truth of the theory but to present it as one prominent account of group agency with important implications for understanding the nature of ICO. With this in mind, we proceed now to an application of Tollefsen’s framework to institutions.

## IV. INSTITUTIONAL AGENCY

It is worth noting at the outset that “institution” can refer to various kinds of social entities, and not all institutions (e.g., the institution of healthcare as such, or gender-classified groups) are aptly regarded as agents.^6^ This is because, on the one hand, some institutions (e.g., the institution of healthcare as such) are not groups but social structures, while others (e.g., the class of all males) are groups lacking the requisite structure for group agency. In such cases, we do not adopt an intentional stance toward the institution in question, since the intentional stance is only adopted when “we assume that the group has a unified perspective—a rational point of view—and that it shares our norms of rationality” (Tollefsen, 2015, 104). Since we focus in what follows on institutional groups of this latter sort, we use “institution” to refer to institutional entities possessing the following two features^7^:

1. *Organization.* An institution is organized if it consists of a group of people with a particular purpose.
2. *Perdurance.* An institution is perdurant if it is not merely ephemeral but involves patterns of behavior and unity over an extended period of time.

In what follows, we use “institution” to refer to perdurant, organized institutions. What is key to note for present purposes is that many healthcare institutions (including most hospitals and private healthcare providers) are perdurant organizational institutions: they have an organized structure and a history.

Some activities and beliefs within institutions are of the sort that lead reasonable observers to attribute them to individuals within the institution, rather than to the institution as such. For instance, hospitals are not regarded as literally performing medical interventions on patients, or having personal relationships with patients; rather, these are actions that are attributed to individual doctors or nurses. In what follows, we focus not on attitudes or actions of individuals *within* institutions (some of which cannot be attributed to institutions as such), but rather on those attitudes and actions that constitute more than simply individual attitudes. For instance, the actions of administrators when speaking on behalf of the institution are instances of what Shadd and Shadd refer to when they observe that “health centres are constituted and animated by such people whose actions *count* as those of the institution” (2019, 209). These actions are not causally generated by the institution, but they nevertheless count as those of the institution.^8^ Put differently, they *constitute* actions of the institution, just as official actions of Israel’s leaders constitute instances of Israel’s group agency, precisely because they are the sorts of institutional activities that lead reasonable observers to adopt an intentional stance toward the institution as a whole.

## V. CONSCIENCE AND CONSCIENTIOUS OBJECTION

Conscience has been defined in different ways, yielding different accounts of the nature of conscientious objection. On some views, conscience is defined as an essentially “private” feature of ethical agents (ACOG, 2007); on other views, conscience is “not a faculty of judging right and wrong that is different from our usual ways of employing our faculties of reason, emotion, and will,” and hence, is not essentially private. Whether or not conscience is a private feature of agents, differing accounts agree in treating conscience as a feature of moral cognition and/or action (or a complex of the two). Such a feature could be “a *faculty* by which agents perceive the fundamental moral structure of universe” (Symons, 2017, 245), or alternatively an intuition (or set of intuitive judgments) (cf. Sulmasy, 2008); or on the other hand, a motivation or emotion that serves to explain a given moral action (Giubilini, 2021). We mention these views simply in order to note that, given any of these definitions of conscience, our foregoing model of institutional agency naturally gives rise to a realist account of institutional conscience. Recall that we are following Tollefsen’s interpretivism, according to which an institution is an agent if it has features that lead observers to adopt the intentional stance in appraising and evaluating it (List and Pettit, 2012). This provides a metaphysical basis for treating the intentional attitudes often ascribed to healthcare institutions (such as cognitive beliefs, motivations, and morally evaluable actions) as evidence for the features constitutive of conscience.

By way of illustration, consider the core commitments that in part define many healthcare institutions. These may be articulated in an institutional mission statement or code of conduct that the institution’s members seek to respect. Catholic healthcare providers in the United States, for example, seek to abide by the *Ethical and Religious Directives for Catholic Health Services*—an ethical code based on the teachings of the Roman Catholic Church (USCCB, 2009). Another case in point would be the institutional values implicit in the history of a given healthcare institution. Catholic hospitals, which were started by religious orders, often see the charism of their founding order as an important part of their institutional identity and mission. These value commitments and histories on which healthcare institutions model their practices are at times put explicitly in terms of group beliefs.^9^ Some would be inclined to resist this claim by seeking to explain institutional activities and beliefs solely in terms of the actions of the individuals which compose those institutions (e.g., the actions and beliefs of hospital board members and administrators, together with the actions of healthcare practitioners who provide services *qua* employees of the hospital). However, according to our interpretivist model, we ought to take the interpretive practices of observers at face value: if reasonable observers ascribe beliefs or convictions to a healthcare institution *as a group*, this reveals an intentional stance that is constitutive of that institution’s status as a group agent, possessing the beliefs and convictions in question.

Whether a given code of conduct constitutes evidence for a hospital’s possessing a conscience depends on which definition of conscience one adopts. For instance, if we assume an intuition-based view of conscience according to which conscience can be identified in a set of deeply held intuitive beliefs, it follows that any deeply held beliefs or convictions ascribed to a healthcare institution as such may constitute evidence not only for the institution’s status as a moral agent, but also for its status as a conscientious agent. Of course, views of conscience, other than the intuition-based account, regards other intentional attitudes (e.g., the faculty or disposition for belief, rather than beliefs as such) as constitutive of conscience. Thus, a range of analyses is required to identify the conscientious status of a given institution. Our present purpose here is not to justify any particular analysis of institutional conscience. Rather, our aim has been to show that, *given* an interpretivist model of group agency, the healthcare institutions that typically feature in debates of ICO possess the intentional and moral attitudes generally regarded as constitutive of conscience-based activity and thought.

This concludes our outline of a model of institutional agency and conscience. We now turn to a discussion of the three arguments against ICO outlined in the introduction of this paper.

## VI. ARGUMENT ONE: INSTITUTIONS ARE DISANALOGOUS TO INDIVIDUALS, AND THEREFORE DO NOT NEED CONSCIENCE PROTECTION

We have argued in the previous section that there is at least one coherent model on which institutions do have a conscience. However, several theorists have argued that institutions do not have a conscience, and therefore need not be afforded conscience protections. In this section, we discuss the basic structure of these arguments, and explain why we believe it to be flawed.

Critics of ICO observe that institutions are fundamentally different to individual human persons. As Spencer Durland observes, “. . . a hospital is not a person; it is a physical structure within which providers give medical care. It does not perform procedures or counsel patients. It does not take lunch hours or vacations” (Durland, 2011, 1659). Building on this idea, critics of ICO note that institutions cannot sustain moral injury in the same way that individual persons could if they were forced to participate in a practice that violates their deeply held moral beliefs (Fletcher, 2016). Moral injury refers to the psychological distress resulting from actions, or the lack of them, which violate one’s moral or ethical code (Litz, 2009). Moral injury is often invoked as a justification for allowing healthcare professionals to opt out of participation in medical procedures to which they have a moral or religious objection (Hanna, 2005). As Wicclair notes: “[u]nlike individuals, hospitals cannot have or lose self-respect or a sense of dignity, and they cannot experience a loss of identity or moral integrity as a harm or injury” (2011, 131).

In light of this, it may be argued, institutions are not morally harmed, even if they are obliged to act in a manner that is contrary to their ethos. Unlike the case of individual conscience, “it seems there is no sound basis for valuing the preservation of institutional identity and integrity” (Wicclair, 2011, 131).^10^ According to this argument, concerns about integrity and moral injury cannot be generalized from individuals to institutions.

Based on this argument, individual personhood and moral integrity are precisely what conscience protections are about. Conscience protections, on this view, require either personhood or a capacity for moral injury. This understanding of conscience protection seeks to rule out the possibility of ascribing a conscience to an institution by pointing out features of individual agency (personhood and individual moral integrity) that are not possessed by institutions. We say more about this understanding below.

Before doing so, it is worth noting that the argument makes significant assumptions about the relationship between ontology and ethics (Suikkanen and Kauppinen, 2019). A key assumption is that the ontological property of having a conscience is morally significant: it bestows a special moral status on its bearer. That is to say, an entity with the property of having a conscience *thereby* possesses distinctive moral rights (conscience-based rights), which it would otherwise fail to have. This assumption is certainly not an innocuous one, and we note that some would regard it as excessive to assume that institutions must literally possess a conscience (of the same sort had by individuals) in order to be granted a right to conscientious objection. Nevertheless, the assumption has the strength of enabling the argument to provide clear, publicly neutral grounds for why institutions do not deserve to be exempted from providing morally controversial procedures on the basis of a conscientious objection.

Even without challenging the foregoing assumption, we believe that our interpretivist model of institutional agency reveals this argument to be problematic in two ways. First, and most importantly, the argument fails to consider sufficiently the impact of metaphysical assumptions about group agency (and the associated notion of group intentional attitudes) on the question of conscience protection. While it is true that there are obvious differences between healthcare institutions and individual doctors, the question is whether the features at issue—namely, the differences that obtain between individual agents and group agents—are the differences that are salient to conscience protections. It is striking that some of the alleged differences mentioned—that only individuals have personhood and can risk losing moral integrity—involve assumptions at the center of contested debates in the metaphysics of groups (List and Pettit, 2012). Those who acknowledge groups as agents have also argued for group personhood. Likewise, those who acknowledge groups as agents also argue that groups, as such, are capable of experiencing moral harm. To suffer harm is often defined as “to be put into . . . a certain sort of bad state or condition” (Hasner, 2008, 451). In the context of groups and institutions, such harms may be nonmoral, as when groups suffer financial losses, or when academic misconduct is spoken of as a “serious harm to the university” (ANU, 2015). However, moral harms occur if the bad state in question leads observers to place moral blame on the group or institution, such as when “acts of abuse” perpetrated by clergy are described as something that not only causes harm to victims but also “damages the Church” with regard to its moral status (Glatz, 2019). Of course, these harms are not experienced by the institution *qua* institution in exactly the way that healthcare practitioners do when made to participate in procedures to which they morally object.^11^ A counter-reply might thus claim that even if institutions can lose moral integrity in a way that constitutes a serious harm, only individuals can *experience* that harm, and that is why conscience protections should be limited to individuals. However, this reply fails to consider that experiences can be accounted for on some models of group agency. On our model, for instance, all group intentional attitudes are understood in a dispositional or functionalist manner: they refer not to inner phenomenological states or brain states, but rather, to tendencies for behavior (Tollefsen, 2015). Thus, it is no more difficult to understand groups as having experiences, than it is to understand groups as having beliefs and actions, so long as they behave in a way that leads observers to attribute the relevant beliefs and experiences to the group. Of course, it might be said in reply that some experiences (of the specifically phenomenological sort) are indeed distinctive to individuals, and it is *these* experiences that are crucial for conscience protection. This position strikes us as an ad hoc maneuver: there are no doubt many features distinctive to individual agents, but more work needs to be done to establish why *these* features are crucial to conscience protections, rather than broader cognitive and behavioral features such as the capacity for deeply held moral beliefs, which are generally associated with conscience.

We have thus far argued that, even if we grant the claim (1) *that the property of having a conscience bestows conscience-based rights*, it does not follow (2) *that only individuals have the property of a conscience*. In particular, we have argued that at least one coherent model of institutional agency yields the conclusion that healthcare institutions do have consciences. This does not suffice as a refutation of (2), but it does show that (2) stands in need of further justification and that critics of ICO thus must articulate their metaphysical assumptions before proceeding. It is worth concluding this section by noting one further way to challenge the argument against ICO considered in this section: even if we also grant (2) to the objector, it still does not follow institutions should not receive conscience protections, because even without a conscience, institutions might still bear rights which are *relevantly similar enough* to those of individual conscience protection rights. One might say that even if institutions do not deserve “conscience protection” (where *conscience protection* is stipulatively limited only to those with the ontological status of being a responsible individual), nevertheless, they deserve something very similar, “conscience protection*,” which should be afforded to any entity which can engage in ethical activity, possess moral beliefs, hold moral responsibility, and experience ethical injury. This kind of protection can be had even by institutions without consciences or conscience protections, and it is not clear why this latter kind of protection is any less deserving of recognition than the former exclusive one (cf. Wicclair, 2011).

Our aim in this section has not been to make the positive case for ICO. Rather, we have sought to show that the argument that institutions lack consciences and thus do not deserve conscience protection is unconvincing. Invoking our model of institutional agency (according to which institutions just as well as individuals possess consciences), we have argued that the burden is on the critic of ICO to defend a metaphysics of group agency that would establish the claim that institutions *lack* consciences, or else cannot be regarded as persons, engage in ethical activity, experience ethical injury, and so on. Despite the real differences between institutions and individual persons, it would appear that we presently have no uncontroversial reason to deny conscience protections to institutions.

## VII. ARGUMENT TWO: PUBLICLY FUNDED HOSPITALS SHOULD PROVIDE SERVICES THAT ARE SAFE AND LEGAL

Another common objection made to ICO is the claim that publicly funded hospitals should provide procedures that are safe and legal.^12^ State support for institutions comes with an expectation that those institutions serve the public interest. Yet ICO allows healthcare providers to pick and choose which duties they choose to fulfill. Many religiously affiliated hospitals in countries such as the United States, Canada, and Australia are the recipients of public funding. Yet, these institutions abide by directives issued by relevant religious authorities that outline the ethical limits of procedures that they can provide. For example, Catholic hospitals in the United States must abide by the *Ethical and Religious Directives for Catholic Health Services* if they are to retain their designation as an official Church institution (USCCB, 2009, 3–5). Similarly, Catholic Hospitals in Australia follow the *Code of Ethical Standards for Catholic Health and Aged Care Services in Australia* (CHA, 2001). As a result, these services do not provide abortions, voluntary sterilizations, emergency contraception, or euthanasia.

Many ethicists argue that publicly funded hospitals have a responsibility to provide a full suite of healthcare services to patients. Scholars argue that an implicit contract is established between the State and healthcare facilities in situations where healthcare facilities are the recipients of public funds. The State rightly expects healthcare institutions to provide care to citizens. Large portions of society, furthermore, are dependent on the public healthcare system to provide for their basic health needs. Publicly funded hospitals must, therefore, fulfill their duties to society and the State and provide appropriate care to patients. As Rhodes and Danziger write: “Society grants hospitals numerous privileges (financial and legal) because of the valuable services that we rely upon them to provide. It is therefore fair to expect them to meet their responsibilities to society” (2018, 52). According to the authors, it is unacceptable for religiously affiliated hospitals to refuse to provide abortion services to women who have a medical need for it. Rather than conscientiously objecting to the provision of these procedures, religiously affiliated hospitals should “get out of the hospital business” (Rhodes and Danziger, 2018, 52).

Similar sentiments have been expressed in public debate about the provision of controversial procedures such as assisted dying. Catholic aged-care providers in Canada have been heavily criticized for refusing to allow residents in their facilities to be assessed for or to receive Medical Aid in Dying (MAiD). In response to complaints about a Catholic hospital in Nova Scotia that refused to provide MAiD, Dalhousie University legal academic Jocelyn Downie told reporters in 2018 that “it’s indefensible to have a publicly funded institution [put] a faith-based filter on the services that are available” (Willick, 2018).

This argument presumes that publicly funded facilities are under an *obligation* to provide procedures that are safe and legal. We call this the *comprehensiveness demand*. In reply, we would like to emphasize that we regard this demand as a genuine obligation on publicly funded facilities, and hence, do not wish to raise any objection to its validity. What we object to is the legitimacy of the argument’s appeal to the comprehensiveness demand in the context of undermining conscience protections. The argument ignores the fact that some religiously affiliated healthcare providers do not believe that abortion and euthanasia are part of healthcare. In the case of terminations of pregnancy, Catholic hospitals reject the view that abortion is basic medical care. These institutions do not include abortion in their conception of reproductive healthcare. Nor do they count euthanasia among the interventions that characterize end-of-life care. Rather, Catholic providers hold that abortion and euthanasia are gravely immoral and should not be provided in any context (USCCB, 2009). In light of this, Catholic healthcare facilities, both private and public, do not provide terminations or help patients in accessing euthanasia services. They see their decision not to provide these services as an exercise of their institutional liberties, rather than as an act of willful discrimination.^13^

At this point, it might be argued that, even if Catholic hospitals have reasons (within the pale of reasonable disagreement) for why they do not violate the comprehensiveness demand, there is nevertheless a need for state-funded facilities to abide by the *State’s* understanding of what constitutes fulfilling the comprehensiveness demand. This point may be clarified by considering the contractual nature of agreements between publicly funded institutions and the State: the State’s agreement to provide a given set of resources is accompanied by expectations as to how those resources are used. If these expectations are reasonable and grounded on publicly available facts, they can be seen as tacit conditions of use underlying the State’s allocation of resources to a given institution. This, in turn, generates an obligation on the part of recipients of public funding: publicly funded institutions ought to abide by the State’s expectations of how public funds ought to be used. We call this the *contractual* demand. Importantly, since the expectations at play in the contractual demand are those of the State, rather than those of the recipient (the healthcare institution), it follows that, even if Catholic providers fulfill the comprehensiveness demand (according to their understanding of comprehensive healthcare), their failure to fulfill the *State’s* understanding of comprehensive healthcare, nevertheless violates the contractual demand.

In reply to this version of the objection, it should be noted that religiously affiliated institutions are open about their mission and identity, and governments choose to fund these institutions cognizant of the services that they do and do not provide. It is difficult to defend the claim, then, that religiously affiliated institutions that receive public funding *are expected by the State*, of necessity, to provide all services that are legal, safe, and desired by patients. Catholic facilities are not funded by governments with an implicit expectation of providing abortion or euthanasia. They are funded with the expectation that they act in accord with their foundational ethos. If anything, Catholic hospitals are honoring the expectation that the State has of them by acting in accord with their basic values and not providing terminations and assistance in dying.

Even so, a critic might still wonder whether states may justifiably fund Catholic hospitals with the expectation that they provide abortions and other legal and professionally accepted medical services. Governments could claim that they have the right to oblige healthcare providers to provide safe and legal services so as to promote the health and well-being of citizens. We should consider, therefore, whether governments may justifiably decide that convenient access to all legal and professionally acceptable services trump an institution’s interest in maintaining its integrity. It is the answer to this question, we submit, that relates to the first objection we discussed, namely, whether institutions have consciences and whether they are harmed if they are forced to act contrary to their conscience. As we have argued, our model of institutional agency makes it difficult for critics of ICO to deny that institutions have consciences other than in ad hoc fashion. Thus, on our model, institutions can experience not only significant harm if forced systematically to violate their institutional ethos: the harm they experience constitutes a violation of conscience. This does not yield a concise answer to the above question; however, our claim is simply that if institutions do have consciences, then the question of whether governments may justifiably undermine an institution’s integrity for the sake of upholding convenient access to a set of public services, is one that should be decided along the same lines as that of whether governments may justifiably undermine an individual’s integrity for the same reasons. If so, then the argument we have considered in this section does not provide evidential weight against ICO over and above the weight of the argument considered in the previous section.

This should suffice for a general overview of the main argument pertaining to the obligations of religious healthcare institutions that receive public funding. We have argued that there is reason to accommodate a variety of reasonable conceptions of what constitutes basic medical care. A critic of ICO could, of course, deny that a Catholic conception of healthcare falls within the pale of reasonable disagreement. We believe that those who make such a claim in the literature fail to offer a principled justification for their view.^14^ However, more importantly, we have further argued that even so, the resulting objection to ICO is not distinctive: if it rests on concerns about the legitimacy of Catholic healthcare ethics, then (no matter how serious the charges are) the prospects for successfully defending ICO from this charge are no worse than the prospects for defending individual conscientious objectors from similar charges. Our model of institutional agency shows that, in the absence of an alternative account of institutions, this parallel holds.

The motivation for many critics of religiously affiliated institutions, however, is not just the fact that healthcare institutions fail to abide by secular standards of comprehensive healthcare, or that they do so using public funding. Rather, some critics argue that some of these institutions, *qua* institutions, occupy a role in the healthcare system such that they can limit access to certain procedures (even if morally controversial ones), to such a severe extent as to be deeply problematic. This consideration appeals to a feature that putatively sets institutions apart from individual doctors (the extent of their reach in the healthcare system as a whole). It is to this consideration that we now turn.

## VIII. ARGUMENT THREE: ICO LIMITS ACCESS TO ESSENTIAL SERVICES

Several theorists have noted that public funded, religiously affiliated hospitals are often the main healthcare providers in certain geographical regions (particularly rural areas). This presents a problem for the provision of services such as abortion, euthanasia, and contraception. As Freedman and Charo have noted,

In communities where the only hospital is religious, or where there is a high level of religious health care saturation, there may be no other options for health care employment or services. As publicly funded entities that control a significant portion of the health care market and resources, religious hospitals, we believe, have a duty to offer comprehensive care. (2018, 2)

There is concern, in other words, that religiously affiliated facilities can in effect control the sorts of services that are provided in communities where healthcare options are limited. Clinicians in these facilities are prohibited from providing interventions that conflict with the ethical code of the organization, and this, in turn, prevents consumers from accessing the care that they desire (or *need*, in the case of medically indicated services).

One commonly discussed example is the provision of abortion for persons in remote communities who cannot access secular healthcare facilities. There are several reported cases of women who have presented at Catholic healthcare facilities in the United States in need of an abortion, and yet have been denied care (Amiri, 2016; Ross, 2017). In other cases, the facility has provided an abortion, but has been subsequently reprimanded by the local Catholic hierarchy (Nelson, 2018). In light of this, it could be argued that religiously affiliated healthcare providers should provide access to services such as abortion where this is necessary to preserve the life and health of patients. At the very least, these facilities should authorize their staff to provide transfers and appropriate information to patients.

This argument identifies a tension between consumers’ access to necessary medical care and the mission and integrity of religiously affiliated healthcare institutions (though there is debate about how much religiously affiliated institutions restrict access). It may be that critics are willing to accept conscientious objection (both for individuals and for institutions) in situations where the position of the person or institution does not severely limit access to care. But where the stakes have been raised and the life or health of a patient is at stake, ICO must give way to patient needs. As Wicclair notes in a discussion about emergency contraception, “Hospitals . . . have obligations to prevent harm to patients, promote patient health, and respect patient autonomy. These obligations set limits to identity- and integrity-maintaining refusals to offer [emergency contraception]” (2011, 132). The basic claim is that the duty of a hospital to prevent patients from experiencing harm trumps the hospital’s right to conscientious objection.

On one interpretation, this is another consequentialist argument against ICO. The argument suggests that the life and health of women is of greater importance than respecting an institution’s mission and values. For context, it is worth situating this objection within the wider debate among consequentialists and deontologists concerning conscientious objection. According to consequentialists, respect for conscience only applies to the extent that it is of net benefit to society. Where the needs of patients or the healthcare system conflict with an institution’s mission and values, institutions must be willing to set their values aside. The bad consequences of denying care to women who are seriously ill, for example, outweighs the good consequences of respecting organizational integrity (Magelssen, 2012).

A deontologist, by contrast, could argue that consequentialist lines of reasoning—in the absence of absolute norms—would in extreme cases allow for the sacrifice of innocent life and blatant discrimination (the sorts of aberrant practices that motivate criticisms of ICO). Utilitarianism seeks to maximize utility, but in some cases this involves an egregious and morally unacceptable infringement of individual liberties—or so a deontologist might argue.^15^ In the context of healthcare, consequentialism would permit sweeping violations of individual and institutional conscience in circumstances where this would be of benefit to patient welfare. Deontologists might on these grounds reject a consequentialist approach to the regulation of conscientious objection. They may deem consequentialism to be too cavalier in its treatment of individual and institutional conscience rights.

We believe that a deontological approach to conscience is, all things considered, a preferable view. However, contributing to the debate between deontologists and utilitarians lies outside the scope of this paper. What we challenge is the claim that conscience should in extreme situations give way to the right to emergency contraception or abortion. Here again, the analogous case of individual conscientious objection makes for useful comparison. Catholic doctors see abortion as a moral evil in which they should play no part, even where it is seen as necessary for the health of a woman. Such a view rejects the very conception of harm and responsibility (and implicit conceptions of necessity) on which the argument is based.^16^ In short, even with deontological norms set to one side, the net outcome of the consequentialist calculus is itself contested by opposing parties on the question of whether essential services such as those mentioned above are a greater good than the harm of violating a person’s liberty and moral integrity. While many commentators believe that abortion is morally permissible in at least in some circumstances, Catholic moral theology deems these practices to be part of a “culture of death” that is deeply harmful to society (John Paul II, 1995). On this view, the consequences of a Catholic doctor’s participation in this culture is catastrophic. On our model of institutional agency, this assessment of the consequentialist calculus can be applied equally to Catholic hospitals as to Catholic doctors, since according to our model, hospitals are just as capable of experiencing the harms associated with the violation of conscience and moral integrity just like doctors.

Yet, the access to essential services argument against ICO need not be framed in consequentialist terms. Rather, it can also be formulated as a deontological argument against ICO. One could argue, for example, that the well-being of patients overrides the integrity-based interests of both individual healthcare practitioners and institutions. Related to this, it could be argued that the fiduciary responsibilities of healthcare practitioners extend to women who are in need of emergency contraception or therapeutic abortions, and that it is morally impermissible for institutions to prevent practitioners from meeting the needs of women in a medical emergency. The right to essential medical care, on this interpretation, would defeat an institution’s right to nonparticipation. Healthcare facilities in remote and rural areas should provide a full suite of safe and legal medical procedures, regardless of their institutional mission and values.

In reply, we note that the calculus and final assessment of the hierarchy of rights and obligations is once again contested ground, between (for example) the ordering of obligations endorsed by Catholic doctors and the ordering of obligations endorsed by secular critics. For instance, a Catholic doctor in a remote area would argue that the duty to ensure that persons in remote and rural areas have access to services such as abortion and contraception is not his to bear, but arguably the State’s. If the State believes that access to abortion, contraception, or assistance in dying is part of essential healthcare, then it is the responsibility of the State to ensure that consumers have access to this care. Religiously affiliated healthcare professionals would argue that it is unethical and counter-productive to oblige them to act contrary to their code of conduct so as to provide these services. Indeed, the European Court of Human Rights (ECHR) has ruled that “States are obliged to organize their health service system . . . to ensure that the effective exercise of freedom of conscience by health professionals . . . does not prevent patients from obtaining access to services to which they are entitled . . .” (ECHR, 2012)^17^

The ECHR made this decision with respect to practitioners who conscientiously object to participation in abortion. The ECHR recognized *both* a right to individual conscientious objection and a right to abortion. It ruled that the State needed to ensure that doctors could freely opt out of participation in controversial procedures *and* that consumers could access the services that they needed.

Some would find both the above argument unconvincing and the consequentialist position described earlier to be more compelling: respect for institutional values is important, but this only goes so far. As Sawicki argues, “in cases of true conflict—such as when a patient seeking emergency treatment is refused care because her treating physician or hospital opposes even lifesaving abortions—the law must strike a balance” (2018, 69). Sawicki suggests that, while providers should not be compelled to provide morally controversial procedures, it would be appropriate to demand that they “face the consequences of [their] choice if [their] refusal causes patient injury that would otherwise be compensable under civil or criminal law” (2018, 69).

Now if a provider has been transparent with the State and with the community about its objection, it is more difficult to criticize the provider for not providing a particular service. In the case of an individual provider, what seems to be most important is that a doctor has been adequately transparent with the community and authorities. Provided he or she has fulfilled their duty, the claim that the doctor has been negligent seems less plausible. Critics may argue that some degree of culpability remains if a patient experiences harm as a result of not being able to access a service. Yet, this would beg the question about who is responsible for the harm. Catholic doctors would deny responsibility for the harm experienced by a patient in the event that they refuse to perform an abortion, for example. One cannot be held morally responsible for failing to engage in wrongdoing, and so the doctors would not consider themselves responsible for subsequent harm experienced by the patient. This is not to deny the doctor’s duty of care for the patient; it is only to claim that the duty of care does not extend to the provision of a termination.

Once again, our model of institutional agency provides an extension of this entire set of arguments in protection of doctor’s rights, to Catholic hospitals *qua* institutions. After all, hospitals, according to our model, are equally bearers of “the effective exercise of freedom of conscience.” Just as the State should arguably organize the availability of healthcare services in such a way that it does not fall to religiously affiliated doctors to provide services to which they object, likewise, the State should organize those services in a way that protects religiously affiliated hospitals from providing such services (Fernandez Lynch, 2015).

As a result, appeals to extreme scenarios in rural or disadvantaged areas do not in and of themselves demonstrate a need for distinctive restrictions on ICO. These scenarios may indicate a need for objecting institutions (and likewise objecting individuals) to be more transparent with the community about the services that it does and does not provide. Critics of ICO have not, however, shown that institutions should be required to provide morally contentious medical procedures in rural or disadvantaged areas.

## IX. CONCLUSION

The aim of this article has been to show that fundamental questions about the metaphysics of institutions require further attention in order to evaluate arguments for and against ICO. To do so, we proposed a framework for an account of healthcare institutions as group agents and bearers of conscience. On the basis of this model, we critically evaluated three arguments against ICO. Each of these arguments is commonplace in public debates about the rights of publicly funded institutions to refuse to provide morally controversial healthcare services. In our responses, we showed how defences of conscientious objection can be extended into defences of ICO. First, just as individual doctors can have conscientious objection rights without denying public funding, likewise the right to exemptions may apply to institutions when they are receiving substantial public funding. While institutions may have the requirement to inform the community of their organizational stance on certain controversial medical procedures, we can follow parallel reasoning to say that this does not go as far as requiring that the institutions provide these services to persons who may otherwise have difficulty in receiving the intervention in question. Our goal has not been to show definitively that institutions have a right to conscientious objection—we have not sought to establish the truth of the model of institutional agency we outline, and we are open to more convincing arguments available for critics of ICO to use. However, we hope to have shown that better arguments require serious engagement with metaphysical assumptions about the nature of institutional agency. For this reason, further scholarly research articulating the metaphysics of institutions would be profitable both for ethicists within the academy as well as for policymakers charged with developing regulations surrounding religiously affiliated healthcare institutions. Approximately one in seven hospital patients in the United States is cared for in a Catholic facility (American Hospital Association, 2018). This is to say nothing of the extensive healthcare services provided for patients by other, non-Catholic, religiously affiliated organizations. Evidently, policy questions surrounding ICO are of increasing relevance to the lives of ordinary citizens. It is our hope that this paper may provide stimulus for detailed and nuanced reflection on the debate on these questions—something that has arguably been missing from public discourse to date.

## Footnotes

1 By “relevant professional associations,” we have in mind peak professional bodies such as the American Medical Association or the British Medical Association. The American Medical Association is currently opposed to active euthanasia, and it is not legal in any US state (in contrast to assisted suicide, which is legal in several states). Yet, it is profitable to consider what the debate about ICO might look like if the Association were to change its position in the future.

2 To be clear, we are claiming that the justification for legal prohibitions on ICO lies in ethical arguments about the responsibilities of institutions to the public. Our aim is to show that these arguments are inadequate and fail to justify legal sanctions on institutions that refuse to participate in the provision of certain procedures.

3 For one exception, see Christopher Tollefsen (2014). Tollefsen’s account of institutional conscience draws on Catholic theology and metaphysical assumptions from the Catholic natural law tradition. Our paper, by contrast, explores institutional conscience without invoking metaphysical or theological assumptions of this sort.

4 Interpretivism has been described as one of two views (the other being functionalism) that “dominate the current debate on group agency” (Strohmaier, 2020, 1).

5 “We might explain the sedentary behavior of a flowerpot by saying that it wants to stay where it is and believes this is the best place for itself” (Tollefsen, 2015, 102). Of course, “in the case of a flowerpot we have a system that does not exhibit a complex pattern of behavior” and so “the intentional stance is not needed,” since a “physical or design stance is more powerful” (Tollefsen, 2015, 102) in explaining the behavior of the entity.

6 In ordinary language, “institution” can refer to various kinds of social entities, from broad social arrangements such as marriage or higher education, to long-standing governmental structures such as the Crown of England (cf. Rust, 2019, 13).

7 These features are drawn from Tollefsen’s (2015) account of corporate groups.

8 We are drawing upon Wolterstorff’s distinction between ‘count’ generation and causal generation. See Wolterstorff (2012).

9 One example is this excerpt from an end-of-life care position statement published by St Vincent’s Health Australia (2017): “St Vincent’s Health Australia believes [. . .] Priority must be given to the policies and resources necessary to ensure all Australians have access to palliative care. Until that priority is met, consideration should not be given to legalising euthanasia and assisted suicide.”.

10 To be clear, Wicclair is not an opponent of ICO. We reference his work only because it provides a lucid summary of the “no conscience” objection to ICO. Wicclair goes on to respond to the objection in a similar way to how we respond to it.

11 One should also consider the possibility of understanding institutional experience in terms of the experience of the CEO or leadership group “counting” as the experience of the institution. In what follows, we assume that the experience is not drawn from an individual in the group, but is a property of the group as such.

12 To be clear, this argument does not focus on a specific subset of publicly funded healthcare institutions. Rather, the commentators in question make a categorical claim about the obligations of *all* public healthcare institutions, independent of the manner in which they are funded. In the United States, for example, the argument would apply to both healthcare facilities owned and operated by an agency of the State as well as to independent healthcare institutions that receive Medicare and Medicaid reimbursement for services provided.

13 To be clear, we do not think that the Catholic conception of healthcare is self-legitimating. Rather, our claim is that the Catholic conception of healthcare falls within the pale of reasonable disagreement (given assumptions about reasonable disagreement made explicit earlier in this paper).

14
Savulescu and Schuklenk (2017), for example, argue that religious objections to medical services like contraception are completely misguided. The authors fail, however, to adequately engage with the literature on reasonableness and reasonable disagreement. It is one thing to say that an argument is wrong; it is another thing entirely to claim that a viewpoint is beyond the pale of reasonableness. A stronger critique of Catholic healthcare ethics is needed—one that shows that this ethical framework is not only misguided but also beyond the bounds of reasonable disagreement.

15 Admittedly, not all consequentialists are committed to the maximization of utility. Consequentialist ethical theory includes but is not limited to utilitarianism.

16 To be clear, Catholic moral theology deems death to be among the gravest of harms. In the case of abortion, however, death would not be considered a harm that an institution could be held responsible for should it refuse to provide the procedure.

17
Fiala and Arthur (2014) claim that the ECHR’s ruling is incoherent. It would be beyond this paper to defend the fundamentality of freedom of conscience as a human right. Suffice it to say that freedom of conscience is typically recognized in charters of rights as right that is of equal importance to other rights that may be used to justify access to abortion. In this respect, the ECHR’s decision is based on a reasonable interpretation of the European Convention on Human Rights.
